# “A Dog Brings Benefits No Matter Where It’s from”: UK Residents’ Understanding of the Benefits and Risks of Importing Puppies from Romania to the UK

**DOI:** 10.3390/ani15152192

**Published:** 2025-07-25

**Authors:** Zoe Belshaw, Elizabeth Youens, Michelle Lord, Rowena M. A. Packer

**Affiliations:** 1 EviVet Evidence-Based Veterinary Consultancy, Nottingham NG2 5HY, UK; z.belshaw.97@cantab.net; 2 Independent Researcher, Gloucester GL15 4NT, UK; eyouens@rvc.ac.uk; 3 Independent Researcher, Bristol BS3 4TW, UK; michelle@co-evolve.co.uk; 4 Department of Clinical Science and Services, The Royal Veterinary College, Hawkshead Lane, North Mymms, Hatfield, Herts AL9 7TA, UK

**Keywords:** puppy, dog, imported, non-endemic disease, zoonotic, veterinary, qualitative, attitudes, welfare

## Abstract

The United Kingdom (UK) is a key market for puppy importation from the European Union (EU), as demand for puppies exceeds domestic supply. Many are illegally imported. Potential welfare risks to the puppies and negative impacts of these on their prospective owners are well documented, but UK residents’ perspectives on the trade are not. This study aimed to use an online survey to capture UK residents’ understanding of the benefits and risks to imported puppies of being imported from Romania for sale in the UK, and the benefits and risks of buying an imported puppy for their prospective owners. Eligible responses were collected from *n* = 7184 participants; 4000 randomly selected free-text comments underwent content analysis. Respondents displayed a limited understanding of the nature and range of risks potentially associated with puppy importation. Worryingly, many viewed an EU-born puppy as equivalent to one bred in the UK; others considered them as “rescued”. Synonyms used for non-endemic diseases that might be carried by the puppies varied widely, suggesting substantial confusion may exist on this topic. The UK public may underestimate the potential risks associated with buying a puppy bred in the EU, which plays into the hands of illegal puppy traders.

## Simple Summary

Each year, many puppies are imported from the European Union (EU) for sale in the United Kingdom (UK), often illegally. The EU puppy trade generates eyewatering profits and huge welfare issues but it is not known how well UK residents understand these, or what potential benefits they might see in owning an EU-born puppy. This study sought to fill that knowledge gap using a survey to capture UK residents’ understanding of the benefits and risks of imported puppies being brought to the UK from Romania, and the benefits and risks for their prospective owners of buying an imported puppy. Valid responses were received from *n* = 7184 respondents; 4000 relevant randomly selected free-text comments were coded. Our findings suggest that UK residents may hold a limited understanding of the nature and range of risks to both imported puppies and prospective owners and may be motivated to acquire one because they perceive them to be equivalent to purchasing a UK-bred puppy. The perceived benefits of “rescuing” an imported puppy appear to align with those described for imported rescue dogs. The UK public may underestimate the potential risks associated with buying a puppy bred in the EU which plays into the hands of illegal puppy traders.

## 1. Introduction

An estimated 10.6–13.5 million owned dogs live in the United Kingdom (UK) [1,2]. The percentage of those which are puppies is not documented but in 2019, prior to the ‘Pandemic Puppy’ boom [3,4,5], 10.3% (*n* = 229,624) of 2.2 million veterinary-visiting dogs were identified to be aged under 1 year old [6]. Demand for puppies in the UK vastly exceeds domestic supply. It is estimated that only 20% of UK puppies come from UK-registered breeders; the remaining 80% are thought to originate from unlicensed domestic breeders, puppy farms or overseas—predominantly from the European Union (EU). Whilst some puppies are legally imported from the EU, often to improve genetic diversity in specific breed lines, it is thought that the majority are illegally imported then sold as if domestically bred [7,8]. Recent changes in puppy purchasing habits are likely to be feeding the illegal puppy trade, including a meteoric rise in online puppy sales during the COVID-19 pandemic [8,9,10,11]; a decrease in prospective owners visiting their puppies in person before purchase [12]; and a substantial increase in the number of owners purchasing puppies with EU Pet Passports [12].

The illegal puppy trade generates both eyewatering profits and huge welfare issues due to the production systems that puppies are typically bred in and the characteristics of breeds commonly produced via these systems. Recent studies of online puppy sales data indicate that dogs with extreme conformations (e.g., brachycephaly and chondrodystrophy) are some of the most commonly advertised puppies for sale in the UK [10,11]. Data suggest that imported puppies also fit this demographic profile, with Dachshunds and French Bulldogs amongst the top five imported breeds [13]. Puppies from the illegal trade may be purposefully bred to exhibit super-extreme physical characteristics such as excessive skin folds and short muzzles which may improve their monetary value but diminish their lifetime welfare due to associated disease risks [14]. Their origins in commercial breeding establishments, or puppy farms, may provide little opportunity for socialisation during this sensitive developmental period, leading to fears, phobias and learning deficits, and may expose them to substantial burdens of disease [15,16,17]. Veterinary care at the point of origin may be minimal or absent, and vaccinations and health certificates may be falsified [13,14]. Illegally imported puppies are typically transported on long overland journeys from countries such as Romania [18] in what can be cramped and dirty conditions without adequate rest [8] and with substantial exposure to disease including zoonoses [8,15]; mortality may be high as a result [14]. Only an average of 5% of dog importation consignments undergo any checks at UK borders [15,19] which facilitates importation of illegally young puppies brought in for commercial sale [13,15,20]. Once in the UK, puppies may be distributed via criminal gangs before onward sale to their eventual owners [14,21].

It is likely that many UK-based owners of EU-bred puppies did not specifically seek to acquire a puppy bred abroad, instead purchasing them via the illegal puppy trade, unaware of their overseas origin [13]. However, a 2020 survey by Dogs Trust suggested that 30% of respondents would consider buying a puppy from an online advertisement even if they thought the puppy might have been illegally imported [22]. Motivators to intentionally acquire a puppy from abroad have been documented. It is typically cheaper to buy an imported pedigree puppy than one bred in the UK [23,24] and a desire to own a puppy with a mutilation such as cropped ears or docked tail has been evidenced as a reason to choose a one bred in a country where this practice is legal [2,25]. Despite these data, it remains unclear what wider public attitudes are towards puppies known to have been bred in the EU for subsequent sale in the UK. For example, a puppy known to be born abroad might be considered as equivalent to a domestically bred puppy or could hold an elevated status more like an overseas ‘rescue’ dog [26,27]. Recent reports [7,13,17] have sought to dissuade prospective buyers from purchasing an EU-bred puppy by highlighting concerns about their welfare, but it remains unclear how much the UK public knows about importing puppies from abroad.

The aim of this study was to use an online survey of UK residents to capture their understanding of the benefits and risks to imported puppies of being brought to the UK and the benefits and risks of buying an imported puppy for their prospective owners.

## 2. Materials and Methods

### 2.1. Ethical Statement

This study was ethically approved by the Social Science Research Ethical Review Board at the Royal Veterinary College (Reference: SR2023-0085). Funding was from the Research England Quality-Related Strategic Priorities Fund. Reporting is as per the relevant aspects of the COREQ checklist [28].

### 2.2. Survey Design and Content

An online survey, “Purchasing puppies survey: would you buy this puppy?” was developed to explore UK residents’ knowledge about, and attitudes towards, purchasing puppies imported from the European Union. Eligible respondents were anyone aged 18 or over living within the UK. Questionnaire development was led by Z.B., refined after discussion with R.P. and piloting. The final questionnaire (see File S1) consisted of 40 multiple-choice and/or free text questions, including four optional questions for current dog owners. Using logic, only the relevant questions were shown to individual participants. Following the initial informed consent statements, no questions were mandatory. Respondents were unable to amend answers on a previous page.

The survey comprised six sections:

1. Participants’ previous and current dog ownership history, as well as their thoughts or plans regarding acquiring a new dog.
2. Questions stemming from a hypothetical online advert for an 8-week-old Cocker Spaniel puppy, examining participants’ perspectives on purchasing the puppy as successive details about its imported background were disclosed, and assessing their understanding of about EU Pet Passport regulations. Some of these results are reported elsewhere [29].
3. Questions centred on a second hypothetical online advert for a 16-week-old Jack-a-Poo (Jack Russell cross Poodle) which was identified to have been born in Romania. The advert read “Gorgeous Jack-a-Poo pups, 16 weeks old and ready to scamper into your hearts. Microchipped, wormed, flea treated, fully vaccinated inc rabies, vet checked. Well socialised with kids and pets. Sold with full papers and feeding guide. Home checks insisted on for all buyers. Distance from your home 5 miles”. Respondents were informed that the puppies were legally imported from Romania aged 15 weeks. Subsequent questions explored participants’ perspectives towards imported puppies, and their awareness and perceptions of risks or benefits to the puppies themselves and/or their prospective owners.
  Pertinent to this paper, respondents were asked whether they could think of any benefits or risks to the welfare of the puppies from being imported to the UK from Romania, and whether they could think of any benefits or risks to a prospective owner’s physical, mental or emotional health from buying an imported puppy. Response options for each were yes, or no. If respondents answered “yes” to any of these four questions, using logic they were given the option to explain their answer(s) in free text.
4. Questions exploring respondents’ concern about, and information sources about, “foreign” diseases. Further questions explored their awareness and knowledge of six specific diseases; these results are reported elsewhere [29].
5. Demographic questions including respondent age, gender, nationality, any history of having lived outside the UK and whether they had worked with, or owned, dogs born in the European Union.
6. Optional further multiple choice and free text questions for current dog owners about where their most recent dog had been sourced and, where relevant, reasons for acquiring a dog born abroad.

### 2.3. Participant Recruitment

SurveyMonkey was used to host the survey, which was disseminated using social media-based snowball sampling between 29th May and 30th June 2023. Study details were shared by a wide range of individuals and organisations (see acknowledgments for full details). Additionally, personalised emails were sent to individuals who had purchased a dog from Pets4Homes in the previous year and to respondents to the research team’s previous surveys [4,5].

The survey was advertised by stating that we wished to understand how the information in puppy adverts affects puppy buying intentions. This strategy aimed to avoid recruitment bias selecting for respondents with particularly strong views or lived experiences related to imported dog or puppy welfare and health.

### 2.4. Content Analysis of Benefits and Risks Free Text Data

Initial data cleaning was performed as described elsewhere [29]. Responses from respondents who had answered any of the four free-text questions about benefits and risks of importation were manually content analysed [30,31] using Microsoft Excel 2016 to manage the data. Authors Z.B. and M.L. independently inductively coded the first 100 free-text responses to each of the four questions (puppy benefit, puppy risk, owner benefit, owner risk). Where respondents provided more than one benefit or risk within each answer, these were individually coded. Based on these 100 responses to each question, initial codes and corresponding code definitions were developed for each question by each author independently. Codes and their definitions were then compared, refined and agreed.

A second round of independent coding was performed by Z.B. and L.Y. on another 100 responses per question to check agreements using the existing code definitions, with results compared and any disagreement again discussed. Substantial further code refinement and combination was performed, before Z.B. and L.Y. independently coded a further random 100 responses per question. Randomization was performed using Excel’s =RAND() function; the random numbers were then sorted into order and the first 100 responses selected. Coding consensus was counted as a percentage agreement for each question. At this point, consensus was acceptable [32] at 92% for puppy benefits, 98% for owner benefits and 90% for puppy risks but unacceptable at 67% for owner risks. The coding framework for owner risks was further refined and a second random 100 responses to this question coded; this time agreement was 97%. Z.B. then coded 1000 random responses per question; final code descriptions and responses per code for each of the four questions are presented below with illustrative quotes. Three quotes are presented where the number of responses per code is ≥10 or where the code is of particular relevance; for codes with <10 responses, two quotes are provided. Spelling and punctuation have been amended in the quotes for readability, but not syntax.

## 3. Results

A total of *n* = 7746 individuals responded to the survey. Of these, *n* = 562 responses were removed during initial cleaning due to: identical IP addresses and responses (*n* = 4); non-UK residents (*n* = 16); respondents aged < 18 years (*n* = 2); and no data entered after the initial questions (*n* = 540). This left *n* = 7184 responses for final analysis. The *n* = for each question is provided with each set of results as no questions reported were mandatory.

### 3.1. Participant Demographics

Most respondents who provided demographic information were female (*n* = 4894/5323; 91.9%). Respondents aged 25–34 years were the most represented (*n* = 1130/5336; 21.2%), followed by 35–44-year-olds (*n* = 1067; 20.0%) and 45–54-year-olds (*n* = 1059; 19.8%). Most respondents lived in England (*n* = 3863/5322; 72.6%), with *n* = 1146 (21.6%) Scottish residents, *n* = 258 (4.9%) Welsh residents, and *n* = 53 (1%) Northern Irish residents. Just under 8% of respondents (*n* = 422/5317) had previously been a permanent resident of a current European Union country, with 7.1% (*n* = 380/5317) having permanently resided outside the European Union (excluding the UK).

#### 3.1.1. Dog Ownership Experience

Current dog owners comprised 82.6% of respondents (*n* = 5982/7184) with only 6.8% (*n* = 488/7184) having never owned a dog. Of the those who were or had been dog owners, 57.8% (*n* = 3839/6651) obtained their newest dog between 2020 and 2023 (range: pre-1995–2023), with 71.7% (*n* = 4802/6690) of owners acquiring a puppy aged < 16 weeks of age. Almost one quarter (24.1%; *n* = 1733/7184) of respondents were considering or currently purchasing a new puppy or dog. This included 43.4% (*n* = 212/488) of respondents who had not previously been a dog owner. Individuals who had travelled outside the UK with a dog comprised 10.5% of respondents (*n* = 560/5329). Most of these (*n* = 394/560; 70%) had previously been resident in a country outside the UK.

Just under one fifth (*n* = 588/4119; 14.3%) of dog-owning respondents who completed the optional questions in section five had owned a dog imported from outside the UK. Of those, 61.9% (*n* = 364/588) had rescued or adopted at their dog(s) from abroad, 28.9% (*n* = 170/588) had bought an overseas-bred dog or dogs, and 13.9% (*n* = 82/588) had fostered at least one dog born outside the UK. A further 7.1% (*n* = 42) were unsure whether their dog had been born within the UK. 

#### 3.1.2. Respondents’ Experience Working with Dogs That Travel to or from the European Union

A total of *n* = 1132/5323 (21.3%) respondents had worked in at least one role with a dog that had originated in the EU. Of these: *n* = 345/1132 (30.5%) had been, or were, veterinary surgeons; *n* = 324/1132 (*n* = 324) veterinary nurses; *n* = 194/1132 (17.1%) had occupied other veterinary roles; *n* = 149/1132 (13.1%) had worked in shelters accommodating dogs from the European Union; *n* = 125/1132 (11%.0%) were dog trainers; *n* = 104/1132 (9.1%) had been involved in showing dogs born in the EU, or had travelled to the EU with UK-born dogs for this purpose; *n* = 52/1132 (4.6%) had occupied a role in boarding kennels; *n* = 41/1132 (3.6%) had taken in foster dogs born in the EU; *n* = 13/1132 (1.1%) had worked in an army role with dogs; *n* = 12/1132 (1.1%) had been dog couriers; *n* = 8/1132 (0.7%) had roles with dogs in security; *n* = 8/1132 (0.7%) worked with dogs in border control; *n* = 5/1132 (0.4%) had worked alongside EU dogs in search and rescue and *n* = 73/1132 (6.4%) had held additional unspecified relevant roles.

### 3.2. Perceived Benefits and Risks to Imported Puppies and Prospective Owners

Overall, *n* = 5839 respondents provided an answer to one or more questions as to whether they could think of benefits or risks to puppies or prospective owners. Benefits to the puppy were considered by *n* = 2227/5839 (41.3%) respondents and risks to the puppy by *n* = 5398/5839 (92.2%) respondents. Benefits to prospective owners were considered by *n* = 2676/5839 (45.8%) respondents, and risks to the prospective owners by *n* = 4336/5839 (74.3%) respondents.

**Figure 1:**
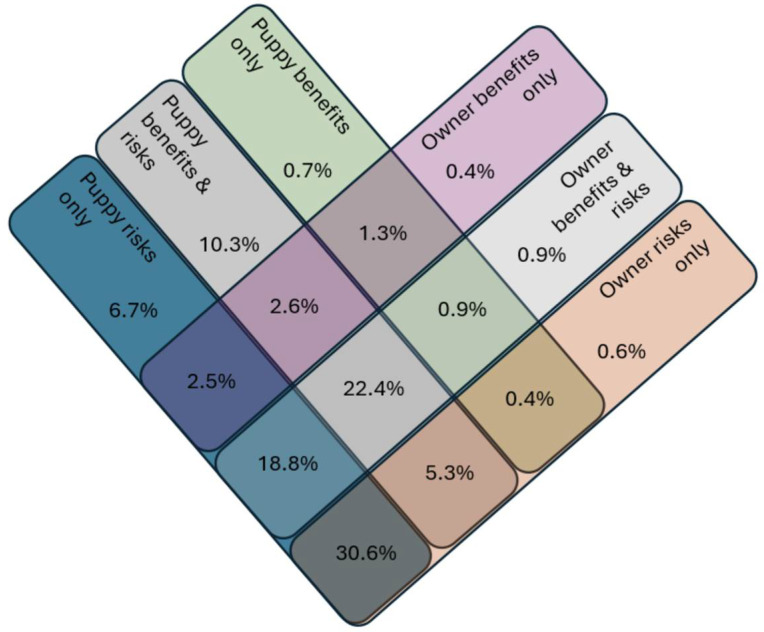
Diagrammatic representation of the proportion of respondents who considered benefits and/or risks to puppies and/or prospective owners (*n* = 5308). Not displayed are *n* = 119 (2.2%) respondents who could not think of neither benefits nor risks to puppy or prospective owners.

A total of *n* = 5308 (90.9%) respondents answered all four questions (see Figure 1 for results overview). Of these, *n* = 1627 (30.7%) could think of just risks to puppy and prospective owner but no benefits, *n* = 1190 (22.4%) could think of benefits and risks to both puppies and prospective owners, *n* = 997 (18.8%) could think of risks to the puppy and benefits and risks to the prospective owners, *n* = 549 (10.3%) could think of just benefits and risks to the puppy, *n* = 354 (6.7%) could think of just risks to the puppy, *n* = 139 (2.6%) could think of benefits and risks to the puppy but only benefits to the owner, *n* = 135 (2.5%) could think of just risks to the puppy and benefits to the prospective owner, *n* = 119 (2.2%) could think of no risks or benefits to either puppy or prospective owner, *n* = 68 (1.3%) could think of just benefits to puppy and prospective owner, whilst *n* = 41 (0.7%) could think of just benefits to the puppy. The remaining *n* = 228 (6.8%) respondents provided other combinations of answers.

A total of 12,747 individual free text responses were collected containing specific benefits and/or risks to puppy and/or prospective owner. The 4000 responses randomly selected for coding originated from *n* = 3286 individual respondents, sampling 56.2% of all valid respondents to the survey and 31.4% of the total free text responses.

### 3.3. Perceived Benefits to the Welfare of Puppies from Being Imported

When asked if they could think of any benefits to puppies’ welfare of being imported from Romania to the UK, *n* = 2227/5785 (38.5%) respondents answered yes; the remaining *n* = 3558/5785 (61.5%) answered no. Of those who said yes, *n* = 1900/2227 (85.3%) provided a free text response; 69 respondents entered free text that they could think of no benefits or provided an answer which could not be interpreted, leaving *n* = 1831 individual respondents. Of the *n* = 1000/1831 (54.6%) responses coded, 37 were descriptions of risks to the puppy, benefits only to the owner, or statement that no benefits could be thought of leaving 963 responses for analysis. From these responses, *n* = 1133 individual perceived benefits were collated and categorised into six codes (see Table 1). Of these respondents, *n* = 795 (82.6%) provided a response which only fitted into one code; *n* = 160 (16.6%) provided a response which fitted into two codes and the remaining *n* = 7 (0.7%) provided a response which fitted into three codes.

The most commonly listed benefits were that the puppy would have a better life in a home in the UK (52.4% responses sampled), and that the puppy would have been removed or rescued from a difficult life in Romania (18.1% responses sampled).

### 3.4. Perceived Risks to the Welfare of Puppies from Being Imported

When asked whether they could think of any risks to a puppy’s welfare from being imported, *n* = 5398/5839 (92.4%) respondents responded yes; the remaining 7.6% responded that they could think of none. Of those who said yes, *n* = 4700/5398 (87%) provided free text. Ten respondents’ free text said they could think of no risks, so these responses were removed leaving *n* = 4690 individual responses. Of the 1000/4690 (21.3%) responses coded, four said “as before”, and two were unintelligible. From the remaining 994 responses, 1367 individual perceived risks were collated and categorised into 11 codes (see Table 2). Of these respondents, *n* = 674 (67.8%) provided a response which only fitted into one code and *n* = 251 (25.5%) provided a response which fitted into two codes. The highest number of codes populated by any individual respondent was four (*n* = 7 respondents; 0.7%).

The most commonly stated risks related to the journey the puppy would have made during importation (*n* = 700; 51.2% responses sampled) and that the puppy’s early life experiences would have been detrimental to their welfare (*n* = 294; 21.5% responses sampled). It was notable that *n* = 69 (5.0%) respondents used a wide range of synonyms to describe diseases which could be brought to the UK from Romania. Specific terms were: additional, different, diseases we don’t have in the UK, exotic, foreign, imported, new, novel, overseas, undetected, unknown, and zoonotic. Non-specific phrases such as “bringing diseases with them” were also coded in this category. Only 14/69 (20.3%) of these responses included the names of specific infectious agents: Brucella (*n* = 8), Leishmania (*n* = 3) and *n* = 1 of each of rabies, heartworm and giardia.

### 3.5. Perceived Benefits to Prospective Owners of Acquiring an Imported Puppy

Asked whether they could identify any risks or benefits to the mental, physical or emotional health of prospective owners of these puppies, *n* = 2676/5399 (49.6%) of those who responded could think of at least one benefit; the rest could think of none. Of these, *n* = 2355 (88.0%) provided a free text explanation. Forty-five used their free text to explain that they could think of no benefits, so when these were removed *n* = 2310 responses remained. Of the *n* = 1000/2310 (44.0%) responses coded, 20 respondents added only risks, and 19 others stated that they could think of none. From the remaining *n* = 961 responses, three codes were developed containing *n* = 1042 coded perceived benefits (see Table 3). Of these respondents, *n* = 880/961 (91.6%) provided a response which was coded into only one code; *n* = 78 (8.1%) respondents provided a response which was coded into two codes and *n* = 2 (0.2%) respondents provided a response which was coded into all three codes.

The most common reasoning provided (*n* = 728; 69.9% responses sampled) was generic benefits from owning a puppy; only *n* = 67 (6.4% responses sampled) contained a benefit that appeared specific to the puppy originating overseas. The content of those responses varied widely and included: *n* = 7 responses suggesting that the puppy would be cheaper, *n* = 7 that the owner would get the exact puppy they wanted, *n* = 4 that they may not have been allowed to adopt from a UK rescue centre, *n* = 2 that the passport would confer travel advantages, and *n* = 2 that the puppy would be particularly likely to bond to the new owners having come from a bad start. It was notable that despite these puppies clearly having been advertised for commercial sale in the UK, *n* = 247 (23.7% responses sampled) suggested that the owner might feel they had rescued the puppy.

### 3.6. Perceived Risks to Prospective Owners of Acquiring an Imported Puppy

Asked whether they could identify any risks to the mental, physical or emotional health of prospective owners of these puppies, *n* = 4336/5422 (80.0%) responded to say they could think of at least one risk to people; the rest could think of none. Of the *n* = 3935/4336 (72.3%) who provided free text, 19 respondents could think of no risks. These responses were removed leaving *n* = 3916 codable responses. Of the *n* = 1000/3916 (25.6%) responses coded, *n* = 6 stated that they had ticked the box in error, *n* = 4 were owner or puppy benefits, and *n* = 2 could not be interpreted. From the remaining *n* = 988 responses, seven codes were developed containing *n* = 1299 coded perceived risks (see Table 4). Of these, *n* = 705 (71.4%) respondents provided a response which fitted into only one code; *n* = 252 (25.5%) respondents provided a response which was coded into two codes and *n* = 29 respondents provided a response which was coded into three codes. The maximum number of codes populated by an individual respondent was four (*n* = 1 respondent).

The most commonly suggested risks to the prospective owner were that they might have negative experiences or feelings associated with owning the puppy due to its background (*n* = 521; 40.1% responses sampled), and that the puppy might be carrying a disease, infection or parasite (*n* = 213; 16.4% responses sampled). Again, a wide range of synonyms were used to describe these diseases (presented as written): additional, contagious, contracted abroad, dangerous, exotic, foreign, foreign-borne, hidden, imported, no longer in the UK, non-endemic, non-native, not known well, novel, preventable, Romanian, transferrable, transmissible, uncommon, unfamiliar, unforeseen, unknown, unseen, zootropic, zoonosed, zoonotic. Fifty-seven specific diseases or causal agents were listed: Brucella (*n* = 35), Leishmania (*n* = 17), rabies (*n* = 2), heartworm (*n* = 1) Babesia (*n* = 1) and mites *n* = 1.

## 4. Discussion

This study represents the first peer-reviewed survey of UK residents’ understanding of human and canine welfare benefits and risks associated with importing puppies to the UK from Romania. It provides useful insights into how individuals might appraise the desirability of owning an imported puppy, and the ethics of this trade, which the UK Government are currently seeking to ban [33]. Respondents could think of more risks than benefits to puppies or people, and of more risks to puppies than risks to people. This appears to contradict the recently documented year-on-year increase in UK-owned puppies acquired with an EU Pet Passport [4], potentially indicating that some owners may not be aware their puppy is imported during the purchase process. Alternatively, if they are aware of their imported status, and understand risks of this, they may consider that benefits to themselves and/or the puppy outweigh these risks. This disconnect potentially fuels the illegal trade if perceived benefits of puppy acquisition via this route are sufficiently high.

A perception that the Romanian puppy would provide equivalent owner benefits to a UK puppy was the most frequent response regarding perceived benefits of acquisition via this route. This is potentially exacerbated by some owners lacking awareness of the future impacts on their puppy’s behaviour and health of being imported [14,16] and the impact of these challenges upon themselves. Over half of respondents who identified risks to a prospective owner (59.9%) did not explicitly identify risks to that person of their puppy being ill, unhealthy, dying, having behaviour problems or needing to be rehomed (e.g., stress, worry, distress or guilt). These are aspects of ‘caregiver burden’ [34] which could negatively impact owner wellbeing over the lifetime of their dog. More effective communication is required to prospective owners about the elevated risks of poor health and behavioural problems in imported puppies compared to those domestically bred, as a deterrent to acquisition.

Perceived benefits to the puppies of being imported largely centred on the UK being a better place to be a dog than Romania in terms of likelihood of finding a secure home, receiving better veterinary care and protection societally under animal welfare laws. The perceptions that Romanian dogs suffer more than those in the UK, are less likely to be adopted and more likely to be killed were also found by Norman et al. [26] to be important motivators for acquiring a Romanian rescue dog. Recent evidence paints a slightly bleaker picture of the UK as a nation of dog lovers than some respondents appeared to believe. Pet rescue centres in the UK have recently been described as “overflowing” [35]. The People’s Dispensary for Sick Animals (PDSA)’s Pet Animal Welfare Report [2] reported that 20% of UK dogs are not vaccinated and 9% of dogs are not registered with a veterinary practice. Furthermore, the Royal Society for Prevention of Cruelty to Animals charity (RSPCA) report having received 61,000 reports of abused or neglected pets in 2024 [36].

In contrast, Romanian living standards for all dogs may not be as universally negative as some respondents suggested (e.g., living in kill shelters or on the streets, and not considered family members). A small sample of pet owners in Romania suggested they perceived dogs to be valuable pets and family members [37]. Despite an estimated 500,000 dogs thought to be living stray in Romania, a report from the European Pet Food Industry Federation FEDIA [38] suggests that 45% of households in Romania owned a dog in 2022, challenging the assumption that all dogs in Romania are street dogs or are in kill shelters. Romania has over 2400 registered veterinary practices [39] and animal welfare legislation passed in 2014 [40] recognises that animals can feel pain and suffer and puts the duty of care onto owners to provide medical attention to pets when necessary. However, these data about Romania were challenging to find from the UK. Negative perceptions of the Romanian dog population as a whole, and Romanian residents’ attitudes towards dogs, may be exacerbated by more visible and striking narratives from the increasing number of international rescue organisations. Norman et al. [26] note that owners of rescue dogs in their study may have been heavily influenced by acting on “emotive images or stories” on social media; some respondents to our survey also alluded to having learnt about Romanian dog welfare from rescue organisations. Accessible, factually accurate data on the status of dog ownership and welfare in Romania could be helpful for individuals seeking to appraise the relative merits of purchasing puppies originating there, or indeed overseas rescue dogs in general.

The range of perceived risks for the puppies associated with importation were comprehensive amongst those respondents sampled. However, the response provided by over two-thirds (67.8%) of respondents sampled populated just one risk code, suggesting that most UK residents may underestimate the range of risks which these puppies might face. Just over half (*n* = 700; 51.5%) of the puppy risk responses coded identified the puppies’ journey across Europe as likely to be detrimental to their welfare, potentially reflecting recent campaigns on this topic [13,15,17] and/or a previous question in our survey related to travel [29]. Some of these responses (21.2%) also identified short- and long-term potential welfare detriments associated with the puppy’s possible origins in a puppy farm. However, only 2% of those responses coded considered that the puppy might not find a suitable home, mirroring the, perhaps unrealistic, finding that a safe new home was the most frequently described benefit for these puppies.

It was notable that more respondents identified a risk to prospective owners from the puppies’ potential for challenging behaviours than that these behaviours might present a welfare risk to the puppies themselves as a reflection of negative underlying emotional states e.g., [41]. Dog behaviours which are problematic to owners are common [42], notably amongst dogs originating from puppy farms and southern European rescue dogs [16,26,43,44], and recognition of their negative impact on caregivers is growing [26,45]. Enhancing prospective owners’ understanding of the potential emotional, time and financial commitments of owning a dog with problem behaviours is urgently needed. This should act as both a deterrent to future acquisition by unprepared owners, and if acquired, may prevent future relinquishment of these dogs in adulthood, given problem behaviours are the most common driver of relinquishment in the UK [46]. Furthermore, communicating to prospective owners that puppies are not a ‘blank slate’ and early life experiences play a crucial role in shaping behavioural development, which will affect their future responses and resilience, is key to managing owner expectations and decision-making.

The risk of the imported puppies carrying infections or diseases into the UK featured in both risks to the puppies and their prospective owners. Awareness of this risk was relatively low with only 16.4% of coded responses to human risks and 5% of the puppy risks coded related to this; this reflects our finding that awareness of specific diseases by the same respondents was generally poor [27]. We were surprised at the wide array of synonyms used to describe these risks, with a total of 31 unique words or phrases counted amongst the coded responses, suggesting that substantial confusion exists as to what these terms mean and/or what the specific risks are. These are important findings given the ongoing risks of travel- and climate-related exotic disease emergence in the UK [19,47,48,49]. We recommend that individuals and organisations seeking to educate the public about these diseases clearly explain the potential risks, and define any overarching terms used.

Almost 70% of coded responses regarding benefits to owners of purchasing an imported puppy suggested that owning a dog imported from Romania would confer no specific advantages over owning a UK bred dog. This mirrors responses to surveys by Norman et al. [26] and PDSA [2] where owners fell in love with, and adopted or purchased, a dog which just happened to come from abroad. This lack of differentiation provides a compelling argument for the cessation of puppy importation.

However, almost one quarter (23.7%) of owner benefit responses coded suggested that the owner of one of these puppies might feel good and/or receive some social approval for having “rescued” the puppies, a motivation not identified in a survey of UK-based Romanian rescue dog owners by Norman et al. [26]. Given the puppy adverts posed to respondents did not state that they were a “rescue”, and were for sale, this reveals underlying perceptions of dogs from overseas implicitly being considered rescued rather than commercially sold. These perceptions potentially bolster this market, as well as creating opportunities for financially motivated “fake rescues” to exploit these feelings.

Adopting a dog has previously been identified as a highly socially desirable method of dog acquisition [50], though individuals in Canada involved in international dog rescue did report some feelings of societal stigma associated with their work [51]. These negative feelings were implicit in some of our responses, suggesting that not everyone considered rescuing puppies to be a virtuous activity, and were reflected in the owner risks where feelings of guilt associated with the acquisition were considered. Nevertheless, “feeling good about rescuing” may represent an important, and hitherto unreported, motivation to acquire an imported puppy, or rescue dog, which should be included as a response option in future surveys of this nature.

It was notable that none of the coded responses referred to a desire to own a dog with a mutilation such as ear cropping, a purchase motivation listed by 9% overseas dog-owning respondents to the 2024 PDSA Paw Report survey [2]. Reasons for this are unclear but may reflect a subtly different respondent population. Other specific benefits listed including the puppies being cheaper and that UK rescue centres might not have allowed the owners to adopt concurred with those described by Norman et al. [26]. This suggests that some motivations to purchase an imported puppy in the context of this study may be shared with motivations to acquire an imported rescue dog [26], which may in turn reflect barriers to acquiring one bred in the UK. 

This research has some recognised limitations; those pertaining to the survey in general are discussed elsewhere [27]. The relatively short free text comments lent themselves well to content analysis. However, respondents included different levels of meaning and explanation within their responses, which made categorization challenging in some instances. Due to the volume of free text, only a percentage of responses in each benefit or risk group were coded [52]; whilst we randomized this sample to avoid any conscious sampling bias, we cannot be certain that these responses were representative of the whole response set.

With the majority of respondents dog owners and > 20% respondents having worked with dogs, of which over 70% had a veterinary role, we also acknowledge that our respondents may have a different level of understanding of the topics raised to the UK population in general. We therefore cannot directly infer that these responses would be reflected in any real purchasing choices made. Additionally, our random sampling method meant we could not appraise individual respondents’ balancing of relative benefits and risks. The three coders involved in this study have a background in clinical canine behaviour (ML), veterinary medicine (EY, ZB) and animal welfare research (ML, EY, ZB); these backgrounds may have impacted the codes developed. As is common in qualitative research dealing with large datasets, e.g., [28], only 10% of the sampled responses were double coded using the final coding framework. Despite the good percentage agreement, it is likely that some responses might have been coded differently by others.

## 5. Conclusions

These data suggest that UK residents may hold a limited understanding of the nature and range of risks associated with purchasing an imported puppy. Prospective owners may be motivated to intentionally acquire a puppy born overseas because they perceive them to be little different to a UK-bred puppy. However, specific perceived benefits of “rescuing” an imported puppy appear to closely align with those described for older imported rescue dogs. We also identify the need for improved clarity on the meanings of terms related to exotic diseases which will continue to present risks to canine and human health even after a puppy importation ban.

## Figures and Tables

**Table 1:** Coded responses to perceived benefits to 16-week-old Jack-a-Poo puppies’ welfare from being imported to the UK from Romania for commercial sale from *n* = 1000 randomly sampled responses. The unique respondent number [RXXX] follows each exemplifying quote.

Code Description	Response Number per Code (*n* = 1133) n (%)	Exemplary Quotes
The puppy will have a better life	594 (52.4%)	Chance of a better life [R2883] A nicer life once on UK soil as long as they are going into suitable homes who have back up and knowledge in helping these dogs [R5336] UK is richer therefore better quality of life [R2070]
The puppy has been removed/rescued from specific aspects of a difficult life in Romania	205 (18.1%)	Romanians are horrible to dogs [R3796] The welfare of puppies in Romania is far from the standards that we have in the UK. Dogs are viewed as family members in UK but that’s not the case in Romania [R825] Would be in kill shelters or on streets in Romania [R1914]
The puppy will be protected by UK animal welfare legislation and attitudes	160 (14.1%)	We have animal welfare laws in place, including the 5 freedoms [R3731] We have a better reputation and infrastructure for caring for animals in the UK [R6613] The UK is a country of dog lovers [R313]
The puppy could access some, or better, veterinary treatment	148 (13.6%)	Better level of vet care [R3327] Someone loving may purchase and take to a vets where it ends up receiving the medical treatment it should have had initially [R4594] Potentially being tested and treated for diseases likely to be contracted abroad, such as leishmaniasis [R5233]
The puppy will have had more health/veterinary checks due to being imported	14 (1.2%)	Health-checks carried out prior to travel [R5579] Has had a rabies vaccination-more immunological cover and likely tapeworm treatment [R5813] Extra vaccines, extra vet checks [R5538]
Other responses which do not fit into the above categories	12 (1.1%)	Exposure to new experiences during the journey, which is an important part of socialisation [R3259] An excellent socialisation process. Puppy meeting plenty of people, fully vaccinated, trained to travel/crate trained [R3083] Brings in bloodlines from abroad that may be healthier than UK dogs [R517]

**Table 2:** Coded responses to perceived risks to 16-week-old Jack-a-Poo puppies’ welfare from being imported to the UK from Romania for commercial sale from *n* = 1000 randomly sampled responses. The unique respondent number [RXXX] follows each exemplifying quote. * See list of synonyms in text.

Code Description	Response Number per Code (*n* = 1367) n (%)	Exemplary Quotes
Travelling from Romania may have been stressful, traumatic or dangerous; the puppy may have been exposed to contagious disease or died through travel	700 (51.2%)	Being in vans for a long time can be scary, especially for young dogs. Probably don’t get much exercise or socialisation [R5383] Risk of disease or injury from transportation, health risk from being kept in hot/cold vehicle, possible dehydration or starvation [R3374] Possible health conditions and stress from flying etc [R3160]
The puppy’s early life experiences are unknown and/or likely detrimental including their breeding, lack of socialisation, being taken from mum too soon, and/or risks of hereditary illness	294 (21.5%)	The risk is you have no idea how the mother dog has been kept and treated and of her current health, is she just a breeding machine? [R5650] Poor socialisation would be my primary welfare concern [R2140] Risk of puppy farming in the overseas territory so undetected long term health issues [R4103]
The puppy might be ill, sick, diseased, poorly treated, stressed, traumatised or dead but the respondent has not specified when/where/why	143 (10.5%)	Stress, anxiety, illness [R4351] Disease, anxiety, stress, fatigue, malnutrition [R330] Malnourished emotionally scarred [R890]
Puppy might be carrying a disease, infection or parasite from abroad *	69 (5.0%)	Rabies and heartworm [R848] Picked up exotic diseases which they brought back with them [R3620] They carry the risk of importing foreign diseases that commonly imported dogs carry like giardia [R4918]
The puppy might have ongoing behaviour changes related to their early life experiences	51 (3.7%)	Travelling puts them in a fearful situation with psychological affects later in life [R2963] Impact to behaviour and development from removal from parents and travel to UK [R6937] Risks lack of socialisation in so many areas, not having lived in a home, not having learned to be independent from siblings after 10 weeks old. I would expect this puppy to be scared of human contact and touch [R2347]
Aspects of the puppy’s background or vaccination status may have been falsified or untraceable–welfare risks implied but not necessarily stated	39 (2.9%)	You don’t know their background or if the documents are legal [R4324] Probably not vaccinated if the paperwork is fake-disease risk [R7701] Are they really socialised or will they be terrified? [R2646]
The puppy might not find a suitable new home in the UK	34 (2.5%)	Not knowing genetic background, or parents could mean they end up in homes where needs cannot be met [R2377] Won’t find a home and end up in a shelter here instead of there [R2841] Possible sale to puppy farmers, dog fight organisers etc. Sale for profit means they may not go to a suitable owner [R5274]
Risks relating to proliferation of the puppy trade for other puppies	14 (1.0%)	Supporting illegal trade [R2803] It also concerns me that there are so many dogs who need rescuing in the UK and from Romania, so breeding more dogs could prevent those who need help from getting it [R3909] It will empower the seller to breed more dogs [R5770]
No specific risks identified but acknowledge they exist	4 (0.3%)	Unknown issues [R936] So many risks [R5740]
Risks no different to any puppy	3 (0.2%)	There is always risk with pets travelling, regardless of age. It is a stressful experience but can be managed [R3131] Same as any dog anywhere-used for breeding/fighting, maltreated [R3605]
Other responses which do not fit into the above categories	16 (1.8%)	Subjected to too many vaccines [R6021] No developed immune system for the diseases in this country [R5945] There will be a level of culture shock they go through for things as small as the difference in weather coming from a warm country to the UK [R661]

**Table 3:** Coded responses to perceived benefits to the new owner of acquiring a 16-week-old Jack-a-Poo puppy which had been imported to the UK from Romania from *n* = 1000 randomly sampled responses. The unique respondent number [RXXX] follows each exemplifying quote.

Code Description	Response Number per Code (*n* = 1042) n (%)	Exemplary Quotes
Generic benefits from having a puppy, but not specifically because it was imported	728 (69.9%)	A dog brings benefits no matter where it’s from [R3412] If they really need a puppy for emotional health, then the origin of the puppy is probably irrelevant [R4339] The benefits of having a new puppy generally could outweigh the concern of buying a puppy from overseas for some owners [R4249]
The owner may feel good from having rescued a puppy	247 (23.7%)	You could feel a sense of pride at having rescued a puppy from Romania [R777] Smugness over having a Romanian “rescue”, the pinnacle of dog ownership apparently [R7348] Giving a rescued dog a better life enhances all aspects of wellbeing [R3655]
Other benefits to the owner specific to the puppy having been imported	67 (6.4%)	Might enable someone who desperately wants or needs a companion to afford one [R1423] Dog was exposed to more things most likely, so the puppy will probably be quick to adapt, saving the owner some levels of stress [R5346] Turned down by other sources when looking for a puppy- some charities set a very high bar when people could actually give a decent home, so this is the only way they can be a pet owner [R7657] Peace of mind that the dog is well bred with no genetic health diseases if research has been done. Peace of mind for breeders that no possibility of close lines [R3791]

**Table 4:** Categorised responses to perceived risks to a prospective owner of acquiring a 16-week-old Jack-a-Poo puppy imported to the UK from Romania from *n* = 1000 randomly sampled responses. The unique respondent number [RXXX] follows each exemplifying quote. * See above text for full list of descriptive terms included in this code.

Code Description	Response Number per Code (*n* = 1299) n (%)	Exemplary Quotes
The owner may find the puppy challenging and/or experience stress, worry, distress or guilt from the puppy being ill, unhealthy, dying, having behaviour problems or needing to be rehomed	521 (40.1%)	A dog with health or behaviour problems can be hard to manage, cut people off from their lifestyle and affect mental health [R7394] The health of the puppy might not be so good and the trauma of losing a pet is horrible [R1378] The risk of physical injury from a semi-feral dog shouldn’t be discounted, but if the dog was just difficult to live with, the owner might feel a failure if he/she had to give up the dog, if they couldn’t cope [R853]
Puppy might be carrying a disease, infection or parasite from abroad *	213 (16.4%)	Risk of contagious diseases [R3140] Imported zoonotic diseases e.g., Babesia canis [R6273] Some vets are reluctant to treat dogs from abroad because of Brucellosis Canis. If the dog is tested in the UK and has a positive result vets would probably want to [put them to sleep] [R7135]
Risks to owner from puppy’s health and/or behaviour problems implied but not specified	190 (14.6%)	Potential health problems with puppy [R1997] If the puppy were to die of an illness shortly after purchase [R4694] Possibly unsociable puppy that has missed important window of opportunity to fit into new home [R5358]
The puppy’s veterinary or behavioural treatment could be expensive	180 (13.9%)	They may have taken on an unhealthy puppy who needs a lot of veterinary attention and the cost of it, which they were probably not expecting or cannot afford [R1564] Unknown parentage could mean health issues so costs more [R4403] If poorly socialised, or has trauma from journey potentially long, and expensive road to getting that pup to be a (bell curve) “normal” adult [R3733]
The owner may feel guilty, stressed or worried about inadequate/false information about puppy, and/or no support from the breeder	180 (13.9%)	Any inconsistencies within the paperwork (i.e., forged information) can be incredibly stressful to deal with [R4480] Stress of looking after a 15-week pup, wondering if it’s got good parents, good health scores, free from disease etc [R4652] The guilt the owner might carry if they found out that the puppy was mistreated, or the mum was used for unethical breeding [R4249]
The risks are no different to a puppy from the UK	7 (0.5%)	Stress of taking care of a pup eg toilet training [R1298] Potential if things go wrong to cause stress and worry-health care bills, training issues (true of a puppy from anywhere) [R5618] Same as buying puppy in UK [R3213]
Other responses which do not fit into the above categories	8 (0.6%)	Communication barrier [R4390] Lots more to consider/check than rabies [R7179]

## Data Availability

Anonymous data, which cannot be traced to any individual, are available upon request for future research from the corresponding author.

## Supplementary Materials

The following supporting information can be downloaded at: https://www.mdpi.com/article/10.3390/ani15152192/s1, File S1: Final complete survey.

## References

[1] UK Pet Food UK Pet Population. https://www.ukpetfood.org/industry-hub/data-statistics-/uk-pet-population-.html.

[2] PDSA Pet Animal Welfare Report. https://www.pdsa.org.uk/media/14944/pdsa_paw-report-2024.pdf.

[3] Siettou C. (2021). Societal Interest in Puppies and the COVID-19 Pandemic: A Google Trends Analysis. Prev. Vet. Med..

[4] Packer R.M.A., Brand C.L., Belshaw Z., Pegram C.L., Stevens K.B., O’Neill D.G. (2021). Pandemic Puppies: Characterising Motivations and Behaviours of UK Owners Who Purchased Puppies during the 2020 COVID-19 Pandemic. Animals.

[5] Brand C.L., O’Neill D.G., Belshaw Z., Pegram C.L., Stevens K.B., Packer R.M.A. (2022). Pandemic Puppies: Demographic Characteristics, Health and Early Life Experiences of Puppies Acquired during the 2020 Phase of the COVID-19 Pandemic in the UK. Animals.

[6] O’Neill D.G., McMillan K.M., Church D.B., Brodbelt D.C. (2023). Dog Breeds and Conformations in the UK in 2019: VetCompass Canine Demography and Some Consequent Welfare Implications. PLoS ONE.

[7] Naturewatch.org BREAKING: Four in Five Dogs and Puppies in the UK Still Come from Unknown Sources. https://naturewatch.org/four-in-five-dogs-and-puppies-in-the-uk-still-come-from-unknown-sources/.

[8] Wilson E., Sopel D. Tricks of the Trade: Exposing the Truth about the Illegal Puppy Trade in the UK. https://media.4-paws.org/1/0/9/c/109ca90267777504402effa9e925ef2bf32d5fcd/Tricks%20of%20the%20Trade%20the%20truth%20about%20the%20illegal%20puppy%20trade%20in%20the%20UK.pdf?_jtsuid=2518717234622088884135.

[9] Anon (2017). New Measures to Tackle “Wild West” World of Online Puppy Sales. Vet. Record..

[10] Paul E.S., Coombe E.R., Neville V. (2024). Online Dog Sale Advertisements Indicate Popularity of Welfare-Compromised Breeds. J. Appl. Anim. Welf. Sci..

[11] Ross K.E., Langford F., Pearce D., McMillan K.M. (2023). What Patterns in Online Classified Puppy Advertisements Can Tell Us about the Current UK Puppy Trade. Animals.

[12] Packer R.M.A., Brand C.L., Belshaw Z., Pegram C.L., Dale F., Stevens K.B., O’Neill D.G. (2023). Is UK Puppy Purchasing Suffering a Long COVID Effect? Ongoing Negative Impacts of the COVID-19 Pandemic upon Puppy Purchase Motivations and Behaviours in 2021. Animals.

[13] Dogs Trust Puppy Smuggling: Puppies Still Paying as Government Delays. https://www.dogstrust.org.uk/downloads/puppy-smuggling-report-2020.pdf.

[14] Maher J., Wyatt T. (2021). European Illegal Puppy Trade and Organised Crime. Trends Organ Crime.

[15] Dogs Trust Puppy Smuggling: A Tragedy Ignored. https://www.dogstrust.org.uk/downloads/puppy-smuggling-report-2017.pdf.

[16] Wauthier L.M., Williams J.M. (2018). Using the Mini C-BARQ to Investigate the Effects of Puppy Farming on Dog Behaviour. Appl. Anim. Behav. Sci..

[17] FourPaws Billion Euro Industry: Why the EU Must Strengthen Regulations to End the Illegal Puppy Trade Now. https://media.4-paws.org/b/5/a/6/b5a69681da94fa61edddf0b00c8a101d2482ba8a/2024-10-29_Billion-Euro-Industry-Report_web.pdf.

[18] Animal Health and Plant Agency Request for Information: Dog Imports. https://assets.publishing.service.gov.uk/media/665f313d0c8f88e868d3356f/FOI2023_16747.pdf.

[19] National Audit Office Resilience to Animal Diseases. https://www.nao.org.uk/wp-content/uploads/2025/06/Resilience-to-animal-diseases.pdf.

[20] British Veterinary Association, British Small Animal Veterinary Association Written Evidence Submitted by The British Veterinary Association and The British Small Animal Veterinary Association (PSO007). https://committees.parliament.uk/writtenevidence/14931/default/.

[21] Maher J.A., Wyatt T. (2019). Rural-Urban Dynamics in the UK Illegal Puppy Trade: Trafficking and Trade in “Man’s Best Friend”. Int. J. Rural. Law Policy.

[22] Pet Advertising Advisory Group Almost a Third of Puppy Purchasers Would Be Willing to ‘Turn a Blind Eye’ to Cruel Smuggling Trade to Get the Dog They Want, According to Dogs Trust. https://paag.org.uk/news/almost-a-third-of-puppy-purchasers-would-be-willing-to-turn-a-blind-eye-to-cruel-smuggling-trade-to-get-the-dog-they-want-according-to-dogs-trust/.

[23] PDSA Pet Animal Wellbeing Report 2023. https://www.pdsa.org.uk/what-we-do/pdsa-animal-wellbeing-report/paw-report-2023/pet-acquisition.

[24] Canine Action UK, C.A.R.I.A.D. Pup Aid Evidence Submitted by Canine Action UK, C.A.R.I.A.D and Pup Aid (PSM0020). https://committees.parliament.uk/writtenevidence/105346/pdf/.

[25] Norris L.J., Pinchbeck G.L., Noble P.M., Radford A.D. (2023). Dogs with Cropped Ears in the UK: A Population-based Study Using Electronic Health Records. Veterinary Record..

[26] Norman C., Stavisky J., Westgarth C. (2020). Importing Rescue Dogs into the UK: Reasons, Methods and Welfare Considerations. Veterinary Record..

[27] Cellan-Jones R. (2024). Sophie from Romania: A Year of Love and Hope with a Rescue Dog.

[28] Tong A., Sainsbury P., Craig J. (2007). Consolidated Criteria for Reporting Qualitative Research (COREQ): A 32-Item Checklist for Interviews and Focus Groups. Int. J. Qual. Health Care.

[29] Belshaw Z., Packer R.M.A. (2025). Knowledge of UK Residents About Importing Puppies from EU Countries. Under Review Preprints.

[30] Kleinheksel A.J., Rockich-Winston N., Tawfik H., Wyatt T.R. (2020). Demystifying Content Analysis. Am. J. Pharm. Educ..

[31] Cunningham M., Wells M. (2017). Qualitative Analysis of 6961 Free-Text Comments from the First National Cancer Patient Experience Survey in Scotland. BMJ Open.

[32] Hallgren K.A. (2012). Computing Inter-Rater Reliability for Observational Data: An Overview and Tutorial. Tutor Quant. Methods Psychol..

[33] UK Parliament Animal Welfare (Import of Dogs, Cats and Ferrets) Bill. https://hansard.parliament.uk/Commons/2025-05-14/debates/27c7ec78-7248-4951-9c88-08594ad59062/AnimalWelfare(ImportOfDogsCatsAndFerrets)Bill.

[34] Spitznagel M.B., Jacobson D.M., Cox M.D., Carlson M.D. (2018). Predicting Caregiver Burden in General Veterinary Clients: Contribution of Companion Animal Clinical Signs and Problem Behaviors. Vet. J..

[35] Loeb J., Gray A. (2024). At Crisis Point: The Challenges Facing the UK’s Rescue and Rehoming Sector. Vet. Rec..

[36] RSPCA Facts, Figures and Myths. https://www.rspca.org.uk/whatwedo/latest/facts.

[37] Rusu A.S., Pop D., Turner D.C. (2018). Geographically Apart, Attitudinally Very Close: A Comparison of Attitudes toward Animals between Romania and Mexico City. People Anim. Int. J. Res. Pract..

[38] FEDIA European Petfood Facts and Figures 2022. https://europeanpetfood.org/wp-content/uploads/2024/06/FEDIAF-Facts-Figures-2022_Online100.pdf.

[39] Federation of Veterinarians of Europe FVE Report on Workforce Shortage of Veterinarians 2024 (REV12). https://fve.org/cms/wp-content/uploads/ap15_VetVision_Shortage_Vets_FYI.pdf.

[40] World Animal Protection Romania. https://api.worldanimalprotection.org/country/romania.

[41] Mendl M., Brooks J., Basse C., Burman O., Paul E., Blackwell E., Casey R. (2010). Dogs Showing Separation-Related Behaviour Exhibit a ‘Pessimistic’ Cognitive Bias. Curr. Biol..

[42] Brand C.L., O’Neill D.G., Belshaw Z., Dale F.C., Merritt B.L., Clover K.N., Tay M.-X.M., Pegram C.L., Packer R.M.A. (2024). Impacts of Puppy Early Life Experiences, Puppy-Purchasing Practices, and Owner Characteristics on Owner-Reported Problem Behaviours in a UK Pandemic Puppies Cohort at 21 Months of Age. Animals.

[43] Baria-Unwalla P., Westgarth C., Buckley L. Adopting a Rescue Dog from Romania: A Cross-Sectional Survey to Identify Factors That Reduce the Likelihood of the Dog Being a ‘Perfect Fit’ for the Adopter’s Household. https://www.research.ed.ac.uk/en/publications/adopting-a-rescue-dog-from-romania-a-cross-sectional-survey-to-id.

[44] Graf J., Kuhne F., Serpell J.A. (2025). Behavioral Traits of Rescue Dogs from Southern and Eastern Europe Rehomed to Germany. J. Vet. Behav..

[45] Barcelos A.M., Kargas N., Assheton P., Maltby J., Hall S., Mills D.S. (2023). Dog Owner Mental Health Is Associated with Dog Behavioural Problems, Dog Care and Dog-Facilitated Social Interaction: A Prospective Cohort Study. Sci. Rep..

[46] Kwan J.Y., Bain M.J. (2013). Owner Attachment and Problem Behaviors Related to Relinquishment and Training Techniques of Dogs. J. Appl. Anim. Welf. Sci..

[47] Wright I., Boyden P., Standbridge A., Elsheikha H. (2025). Importation of Dogs: Animal and Public Health Risks and Possible Solutions. Companion Anim..

[48] Wright I., Whitfield V., Hanaghan R., Upjohn M., Boyden P. (2023). Analysis of Exotic Pathogens Found in a Large Group of Imported Dogs Following an Animal Welfare Investigation. Vet. Record..

[49] Mackenzie J.S., Jeggo M. (2019). The One Health Approach—Why Is It So Important?. Trop. Med. Infect. Dis..

[50] Bir C., Widmar N., Croney C. (2017). Stated Preferences for Dog Characteristics and Sources of Acquisition. Animals.

[51] von Rentzell K.A., Bratiotis C., Protopopova A. (2024). “It’s My Calling”, Canadian Dog Rescuers’ Motives and Experiences for Engaging in International Dog Rescue Efforts. PLoS ONE.

[52] Kim H., Jang S.M., Kim S.-H., Wan A. (2018). Evaluating Sampling Methods for Content Analysis of Twitter Data. Soc. Media Soc..

