# Supplementary material for: “A Dog Brings Benefits No Matter Where It’s from”: UK Residents’ Understanding of the Benefits and Risks of Importing Puppies from Romania to the UK
Source: Animals (Basel). 2025 Jul 25;15(15):2192. doi: 10.3390/ani15152192 (PMC12345484; doi:10.3390/ani15152192)
Supplement: Supplementary file 1 [file animals-15-02192-s001.zip › animals-3715753-supplementary.pdf]

Royal Veterinary College
University of London

Purchasing Puppies survey: Would you buy this puppy?

Introduction

What is this survey about?

This survey aims to explore how people in the UK might find a new puppy, and the decisions they make when choosing which puppy they might purchase. The results will guide educational campaigns, policies and strategies that will help to improve the welfare of puppies, and the experiences of puppy purchasers in the future.

Who is conducting this research?

We are a team of experienced researchers based at the Royal Veterinary College in London, interested in exploring the health and welfare of the UK dog population.

Who can take part in this survey?

Anyone over the age of 18 and resident in the UK can take part. We would like to hear from you whether or not you have ever owned a dog.

What would you like me to do?

First, we would like you to answer a few questions about whether or not you have owned a dog. Then, we would like you to look at two fictional advertisements that offer puppies for sale before answering some questions based on them. The survey finishes with a few questions about you and, if you would like to tell us, the option to answer a couple more questions about any dogs you might have owned.

We are not asking for any personal or identifying details, so all your responses are completely anonymous. Completing the survey should only take about 12 minutes.

What will happen to my information?

Your information will be stored securely by the Royal Veterinary College in accordance with general data protection regulations (GDPR). Only the researchers undertaking this study (Dr. Rowena Packer, Dr. Zoe Belshaw and their immediate team) will have access to your answers. We may include some of your written responses in publications or at relevant conferences but since we are not collecting any data to identify you, only you might be able to recognise what you wrote.

Anonymous data, which cannot be traced to any individual, may be made freely available for future research and learning via the RVC Research data storage facility, and in association with subsequent publications

Can I change my mind and withdraw my answers?

You are free to leave the survey at any point. As your responses are fully anonymous, we will not be able to identify your response, so you will not be able to request that your response is withdrawn from the overall dataset once your answers are submitted.

Who is funding this research?

This study is funded by Research England Quality-Related Strategic Priorities

Funding.

What if taking part raises concerns?
Resources on issues raised are provided at the end of the survey. This study has received ethical approval from the Social Science Research Ethical Review Board at the Royal Veterinary College (ethics approval reference: URN SR2023-0085). If you have any questions about the study, please contact Dr. Rowena Packer (rpacker@rvc.ac.uk).

* 1. **I wish to take part in this research and confirm that:**

- ☐ I am 18 years old or over
- ☐ I am a resident of the UK
- ☐ I have read and understood the above information and give consent for my answers to be used for this research study and in any resulting publications
- ☐ I give permission for the anonymous data that cannot be traced to any individual to be deposited in the RVC research data storage facility and made available in association with subsequent publications so it can be used for future research and learning

RVC Royal Veterinary College University of London

Purchasing Puppies survey: Would you buy this puppy?

These first questions ask about your own experience with dogs. Please remember, we would like you to take part even if you have no experience with dogs.

2. Do you currently own a dog, or dogs?

- ○ Yes
- ○ No

RVC Royal Veterinary College University of London

Purchasing Puppies survey: Would you buy this puppy?

3. Have you ever owned a dog, or dogs, while living in the UK?

◯ Yes

◯ No

RVC Royal Veterinary College University of London

Purchasing Puppies survey: Would you buy this puppy?

4. In what year did you most recently acquire a dog or puppy?

5. What age was your most recent dog or puppy when you brought them home?

◯ Under 16 weeks of age

◯ Over 16 weeks of age

◯ Not sure/can't remember

RVC Royal Veterinary College University of London

Purchasing Puppies survey: Would you buy this puppy?

6. Are you currently considering, or in the process of, getting a new dog or puppy?

◯ No

◯ Yes, I/we are currently considering it but have not started looking

◯ Yes, I/we are currently looking

◯ Yes, I/we have started the buying/adoption process

◯ Other (please specify)

RVC Royal Veterinary College University of London

Purchasing Puppies survey: Would you buy this puppy?

We will now ask questions about puppies that you might consider buying based on two fictional adverts from reputable UK pet-selling websites.
Don't worry if you think you might not know the answers. We are interested in your thoughts and opinions even if you have no current interest in buying a puppy.

Advert 1.
Please read this fictional advert and answer the questions below.

Adorable 8 week old Cocker Spaniel puppies ready to go to their forever families. Microchipped, vet checked, and 1st vaccines done. Flea and worming up to date. Mum and Dad much loved pets, socialised with kids and other dogs. Available to view and take home now. Will only sell to the best homes. Distance from your postcode: 5 miles.

7. **You are interested in buying an 8 week old Cocker spaniel puppy. You can easily get to the location to meet the seller and see the puppies, and they are within your price range.**

Based on the information in the advert above, would you consider arranging to see these puppies with a view to buying one?

- ◯ Yes
- ◯ No
- ◯ Not sure

Please tell us the reason(s) behind your answer.

Royal Veterinary College
University of London

Purchasing Puppies survey: Would you buy this puppy?

This is the same advert. Please read it and answer the questions below.

Adorable 8 week old Cocker Spaniel puppies ready to go to their forever families. Microchipped, vet checked, and 1st vaccines done. Flea and worming up to date. Mum and Dad much loved pets, socialised with kids and other dogs. Available to view and take home now. Will only sell to the best homes. Distance from your postcode: 5 miles.

8. **You call the person selling the puppies to get some more information. During the course of the conversation, they tell you that these puppies each have an EU (European Union) Pet Passport.**

Does knowing that the puppies have an EU Pet Passport change whether you are interested in buying one?

- ◯ Yes, the passport makes me <u>more</u> likely to buy one
- ◯ Yes, the passport makes me <u>less</u> likely to buy one
- ◯ No change – I would still consider buying one
- ◯ No change – I would still not buy one
- ◯ Not sure

Please tell us the reason(s) behind your answer.

Royal Veterinary College
University of London

Purchasing Puppies survey: Would you buy this puppy?

9. Which of the following do you believe are correct about EU Pet Passports? Please answer each question, even if you aren't familiar with EU Pet Passports.

True or false, an EU Pet Passport...

	True	False	Not sure	Don't know
Can be issued in the UK	◯	◯	◯	◯
Means that a puppy or dog can travel from the UK to European Union countries without extra paperwork	◯	◯	◯	◯
Means that a puppy or dog has been vaccinated against rabies	◯	◯	◯	◯
Means that a puppy or dog has had all their standard UK vaccinations	◯	◯	◯	◯
Means that a puppy or dog has tested negative for infectious diseases	◯	◯	◯	◯
Means that a puppy or dog was born in the European Union	◯	◯	◯	◯
Means that the puppy or dog is registered with a Kennel Club in the European Union	◯	◯	◯	◯

This is the same advert. Please answer the questions below.

Adorable 8 week old Cocker Spaniel puppies ready to go to their forever families. Microchipped, vet checked, and 1st vaccines done. Flea and worming up to date. Mum and Dad much loved pets, socialised with kids and other dogs. Available to view and take home now. Will only sell to the best homes. Distance from your postcode: 5 miles.

10. **EU Pet Passports stopped being issued in the UK in 2021, after Brexit. It is now likely that any puppy in the UK with an EU Pet Passport has been born in, and imported from, the European Union. These puppies were therefore almost certainly born outside the UK.**

These puppies are still available for sale. Has the information that they were born abroad affected whether you would still consider buying one?

- ◯ Yes, I would now be more likely to buy one
- ◯ Yes, I would now be less likely to buy one
- ◯ No change – I would still consider buying one
- ◯ No change – I would still not buy one
- ◯ Not sure

Please add any comments that you would like to about your decision.

11. Would your decision have been different if the advert had clearly stated they had been born abroad?

◯ Yes

◯ No

◯ Not sure

Please explain your answer

12. Look at the information in the advert again. Do you believe that these puppies were imported legally for sale into the UK?

◯ Yes, legally imported

◯ No, illegally imported

◯ Not sure

◯ Don't know

RVC Royal Veterinary College University of London

Purchasing Puppies survey: Would you buy this puppy?

This is the same advert. Please answer the questions below.

> Adorable 8 week old Cocker Spaniel puppies ready to go to their forever families. Microchipped, vet checked, and 1st vaccines done. Flea and worming up to date. Mum and Dad much loved pets, socialised with kids and other dogs. Available to view and take home now. Will only sell to the best homes. Distance from your postcode: 5 miles.

13. **EU Pet Passports can only legally be supplied within the EU to puppies over 15 weeks of age. These 8 week old puppies must therefore have been illegally imported for sale in the UK.**

Of all the puppies and dogs brought into to the UK in 2020, what percentage do you believe are estimated to have arrived illegally? Tick your best guess.

- ◯ 0%
- ◯ 1%
- ◯ 3%
- ◯ 5%
- ◯ 10%

14. Has the information that these puppies were illegally imported changed whether you would consider buying one? It would NOT be illegal to buy and own one of these puppies.

◯ Yes, I would now be more likely to buy one

◯ Yes, I would now be less likely to buy one

◯ No change – I would still consider buying one

◯ No change – I would still not buy one

◯ Not sure

Please tell us the reason(s) for your answer.

15. If you or someone else bought one of these illegally imported puppies, would you think of that puppy as having been “rescued”?

◯ Yes

◯ No

◯ Not sure

◯ Don’t know

Please tell us the reason(s) behind your answer.

RVC Royal Veterinary College University of London

Purchasing Puppies survey: Would you buy this puppy?

16. Importing these puppies for sale was illegal, and the seller is breaking the law. Who do you believe this seller could be reported to for action to be taken? Tick all that you think might apply.

- ☐ Police
- ☐ RSPCA/SSPCA/USPCA
- ☐ Trading standards
- ☐ Website listing the sale
- ☐ Local council
- ☐ UK border force
- ☐ A veterinary practice
- ☐ Don't know
- ☐ Other (please specify)

RVC Royal Veterinary College University of London

Purchasing Puppies survey: Would you buy this puppy?

Advert 2.

Please read the second fictional advert below from a reputable pet-selling website and answer the questions. Again, imagine these puppies are the breed and age you are looking for.

Gorgeous Jack-a-Poo pups, 16 weeks old and ready to scamper into your hearts! Microchipped, wormed, flea treated, fully vaccinated inc rabies, vet checked. Well socialised with kids and pets. Sold with full papers and feeding guide. Home checks insisted on for all buyers. Distance from your postcode: 5 miles.

17. **You call to find out more about these puppies and the seller tells you they were imported from Romania at 15 weeks of age. Their import paperwork confirms that they have been legally imported, are vaccinated against rabies, and have had a veterinary health check.**

You have been looking for a 16 week old Jack-a-Poo for a while, and this litter are within your price range. Would you consider arranging to visit these puppies with a view to buying one?

◯ Yes

◯ No

◯ Not sure

Please tell us the reason(s) behind your answer.

RVC Royal Veterinary College University of London

Purchasing Puppies survey: Would you buy this puppy?

This is the same advert. Please answer the questions below.

Gorgeous Jack-a-Poo pups, 16 weeks old and ready to scamper into your hearts! Microchipped, wormed, flea treated, fully vaccinated inc rabies, vet checked. Well socialised with kids and pets. Sold with full papers and feeding guide. Home checks insisted on for all buyers. Distance from your postcode: 5 miles.

18. How do you think these puppies were most likely to have travelled from Romania?

- ◯ Plane
- ◯ Car
- ◯ Lorry
- ◯ Van
- ◯ Don't know
- ◯ Other (please specify)

19. If you or someone else bought one of these puppies, legally imported from Romania, would you think of that puppy as having been "rescued"?

- ◯ Yes
- ◯ No
- ◯ Not sure
- ◯ Don't know

Please tell us the reason(s) behind your answer

Royal Veterinary College University of London

Purchasing Puppies survey: Would you buy this puppy?

This is the same advert. Please answer the questions below.

Gorgeous Jack-a-Poo pups, 16 weeks old and ready to scamper into your hearts! Microchipped, wormed, flea treated, fully vaccinated inc rabies, vet checked. Well socialised with kids and pets. Sold with full papers and feeding guide. Home checks insisted on for all buyers. Distance from your postcode: 5 miles.

20. Can you think of any benefits or risks to these puppies' welfare (physical, mental or emotional health) from having been imported to the UK from Romania?

	Yes	No
Benefit(s) to puppies' welfare from being imported to the UK	○	○
Risk(s) to puppies' welfare from being imported to the UK	○	○

Royal Veterinary College
University of London

Purchasing Puppies survey: Would you buy this puppy?

This is the same advert. Please answer the questions below.

Gorgeous Jack-a-Poo pups, 16 weeks old and ready to scamper into your hearts! Microchipped, wormed, flea treated, fully vaccinated inc rabies, vet checked. Well socialised with kids and pets. Sold with full papers and feeding guide. Home checks insisted on for all buyers. Distance from your postcode: 5 miles.

21. What did you think these benefits and/or risks were for these puppies' welfare from being imported to the UK from Romania?

Benefit(s) to puppies' welfare from being imported to the UK

Risk(s) to puppies' welfare from being imported to the UK

22. Can you think of any benefits or risks that might occur to the physical, mental or emotional health of an owner who buys a puppy imported to the UK from Romania?

	Yes	No
Benefit(s) to new owner's physical, mental or emotional health	○	○
Risk(s) to new owner's physical, mental or emotional health	○	○

RVC Royal Veterinary College University of London

Purchasing Puppies survey: Would you buy this puppy?

23. What were the benefits and/or risks that you could think of that might occur to the physical, mental or emotional health of an owner who buys a puppy imported to the UK from Romania?

Benefit(s) to new owner's physical, mental or emotional health

Risk(s) to new owner's physical, mental or emotional health

Royal Veterinary College
University of London

Purchasing Puppies survey: Would you buy this puppy?

24. What, if any, is your level of concern about imported puppies and dogs bringing diseases into the UK that are not currently found here?

◯ Not at all concerned

◯ A little concerned

◯ Very concerned

◯ Not something I had thought about

Royal Veterinary College
University of London

Purchasing Puppies survey: Would you buy this puppy?

25. Where have you heard about the risk of imported diseases in imported puppies and dogs? Tick all that apply.

- ☐ Training for my job e.g. veterinary medicine, veterinary nursing, rescue centre
- ☐ Media e.g. BBC News, radio, newspapers
- ☐ Social media
- ☐ Information/campaigns from animal charities e.g. Dogs Trust, RSPCA, PDSA
- ☐ Information/campaigns from the UK Goverment/ DEFRA/ DAERA
- ☐ Own experience e.g. travelled with a dog, own an imported dog
- ☐ Experiences of, or discussion with, family or friends
- ☐ Not sure/can't remember
- ☐ Other (please specify)

26. **There is growing concern about imported dogs and puppies bringing new diseases into the UK. The infectious diseases listed below can be associated with dogs legally and illegally imported into the United Kingdom.**

Have you heard of any of these?

	Yes, heard of it	No, not heard of it	Not sure
Rabies	○	○	○
Brucellosis (Brucella)	○	○	○
Leishmaniasis (Leishmania)	○	○	○
Babesiosis (Babesia)	○	○	○
Ehrlichiosis (Ehrlichia)	○	○	○
Hepatozoonosis (Hepatozoon)	○	○	○
Heartworm (Dirofilaria)	○	○	○

Royal Veterinary College
University of London

Purchasing Puppies survey: Would you buy this puppy?

27. Do you believe an infected, imported puppy could infect a human in the UK with any of these diseases (including tick transmission)? Please answer even if you don't know anything about this topic.

	Yes, infected puppy could infect a human in the UK	No, infected puppy could not infect a human in the UK	Not sure	Don't know
Rabies	◯	◯	◯	◯
Brucellosis (Brucella)	◯	◯	◯	◯
Leishmaniasis (Leishmania)	◯	◯	◯	◯
Babesiosis (Babesia)	◯	◯	◯	◯
Ehrlichiosis (Ehrlichia)	◯	◯	◯	◯
Hepatozoonosis (Hepatozoon)	◯	◯	◯	◯
Heartworm (Dirofilaria)	◯	◯	◯	◯

28. Do you believe an infected, imported puppy could infect another dog in the UK with any of these diseases (including tick transmission)? Please answer even if you don't know anything about this topic.

	Yes, could infect another dog in the UK	No, could not infect another dog in the UK	Not sure	Don't know
Rabies	◯	◯	◯	◯
Brucellosis (Brucella)	◯	◯	◯	◯
Leishmaniasis (Leishmania)	◯	◯	◯	◯
Babesiosis (Babesia)	◯	◯	◯	◯
Ehrlichiosis (Ehrlichia)	◯	◯	◯	◯
Hepatozoonosis (Hepatozoon)	◯	◯	◯	◯
Heartworm (Dirofilaria)	◯	◯	◯	◯

RVC Royal Veterinary College University of London

Purchasing Puppies survey: Would you buy this puppy?

Last few questions....

We would finally like to ask a few questions about you, then the survey is complete and you will find a page of resources that might be useful.

29. What is your age?

- ◯ Under 18
- ◯ 18-24
- ◯ 25-34
- ◯ 35-44
- ◯ 45-54
- ◯ 55-64
- ◯ 65+
- ◯ Prefer not to say

30. What is your gender?

- ◯ Male
- ◯ Female
- ◯ Other
- ◯ Prefer not to say

31. Where do you currently live?

- ◯ England
- ◯ Wales
- ◯ Scotland
- ◯ Northern Ireland
- ◯ Other, please specify

32. Have you ever been a permanent resident of another country? Tick all that apply.

☐ Yes, country currently within the EU

☐ Yes, country currently outside the EU

☐ No

33. Have you ever taken a dog or puppy outside the UK [England, Wales, Scotland, Northern Ireland], including on holiday?

◯ Yes

◯ No

◯ Not sure/can't remember

34. As part of your job, have you ever worked with dogs that travel to or from the European Union?

◯ Yes

◯ No

◯ Not sure

RVC Royal Veterinary College University of London

Purchasing Puppies survey: Would you buy this puppy?

35. In what role(s) have you worked with dogs that travel to or from the European Union? Tick all that apply.

- ☐ Veterinary surgeon seeing imported/exported pets in a clinical setting
- ☐ Veterinary surgeon with OV status for small animal export and travel
- ☐ Veterinary surgeon in another role
- ☐ Veterinary nurse
- ☐ Other role within a veterinary practice
- ☐ Shelter/rehoming centre, charity or network
- ☐ Dog fostering
- ☐ Boarding/quarantine kennel
- ☐ Dog breeding
- ☐ Dog showing/competing
- ☐ Dog courier/travel business
- ☐ Armed forces
- ☐ Security
- ☐ UK Border Control
- ☐ Search and rescue
- ☐ Dog trainer/behaviourist
- ☐ Other (please specify)

36. Where did you hear about this survey? Tick all that apply

- ☐ Twitter
- ☐ Facebook
- ☐ Reddit
- ☐ Instagram
- ☐ TikTok
- ☐ Pets4Homes
- ☐ Pandemic Puppies email
- ☐ Newsletter or magazine
- ☐ Word of mouth
- ☐ Not sure/can't remember
- ☐ Other (please specify)

37. Finally, if you own or have owned a dog, and would be happy to answer a few more questions about your own experiences, please tick "yes". Otherwise, "no" will take you to the end of this survey.

- ◯ Yes, I have owned a dog and would be happy to answer a few more questions
- ◯ No, I don't want to answer any more questions

RVC Royal Veterinary College University of London

Purchasing Puppies survey: Would you buy this puppy?

38. **Thank you for agreeing to answer a few more questions.**

Thinking about the dog or puppy that you most recently acquired, where did you get them from?

- ◯ Private breeder or seller I knew already, or was personally recommended to me
- ◯ Private breeder or seller I found on a pet sales website e.g. Pets4Homes, ChampDogs
- ◯ Private breeder or seller I found on a general sales website e.g. Gumtree, Preloved, FreeAds
- ◯ Private breeder or seller I found via the Kennel Club's 'Find A Puppy' search
- ◯ Private breeder or seller I found through social media
- ◯ Rescue or rehoming organisation/charity for UK dogs e.g. Dogs Trust, local rescue centre
- ◯ Rescue or rehoming organisation/charity specifically for dogs from abroad
- ◯ Directly from another seller e.g. previous owner
- ◯ Gifted or inherited from a friend or relative
- ◯ Bred myself
- ◯ Pet shop
- ◯ Brought them to the UK with me when I moved here from abroad
- ◯ Not sure/can't remember
- ◯ Other (please specify)

39. Whilst living in the UK, have you ever bought, rescued/adopted, or fostered puppies or dogs that were born outside the UK [where the UK means England, Wales, Scotland and Northern Ireland]? Please tick all that apply, including if this was your most recent puppy as above.

- ☐ No
- ☐ Yes, bought
- ☐ Yes, rescued/adopted
- ☐ Yes, fostered
- ☐ Not sure
- ☐ Don't know

Royal Veterinary College
University of London

Purchasing Puppies survey: Would you buy this puppy?

40. Think about your most recently acquired dog or puppy that was born outside the UK. Did you specifically want a dog/puppy born outside the UK?

- ◯ Yes, I wanted a dog/puppy born outside the UK
- ◯ No, I would have preferred a UK bred dog/puppy
- ◯ I didn't mind where they came from
- ◯ I hadn't planned to get another dog/puppy then I saw/heard about this one...
- ◯ Not sure/can't remember

Other (please specify)

41. What led to you acquiring that puppy or dog that had been born outside the UK? Tick all that apply.

- [ ] Didn't mind the dog/puppy being born abroad
- [ ] Dogs and puppies born abroad have suffered more and I wanted to help one
- [ ] Wanted a dog/puppy with cropped ears
- [ ] Wanted a dog/puppy with a docked tail
- [ ] Wanted a specific breed, colour or type of dog that wasn't available in the UK
- [ ] Time pressure to get a new dog/puppy
- [ ] Price
- [ ] Previous good experience of owning a puppy or dog from abroad
- [ ] Breeder/rescue organisation recommended by friends or family
- [ ] Wanted a dog/puppy from a specific breeder or breed line abroad
- [ ] Wanted a rescue dog/puppy but UK-based rescue centre(s) would not give me one
- [ ] Wanted a rescue dog/puppy but could not find one I liked that was born in the UK
- [ ] Came across this particular dog/puppy and wanted it
- [ ] Wasn't aware they were born abroad when I started the purchase/rehoming process
- [ ] Inherited/gifted to me
- [ ] Other (please put your reason(s) below)

Please tell us more about your choice to acquire a dog/puppy born outside the UK.

Purchasing Puppies survey: Would you buy this puppy?

Exit and further information

Thank you very much for completing our survey; your time and effort is very much appreciated.

We understand that this survey may have raised some concerns, and therefore we have put together some information below that we hope will be helpful.

Thinking about buying a dog in the future? Avoid getting Petfished and use the Puppy Contract

The Petfished public information campaign is a Government initiative from the Department for Environment, Food and Rural Affairs (DEFRA) in England that aims to alert the public to unscrupulous breeders of puppies and kittens. Petfishing is where unscrupulous sellers pretend that the puppy they're selling you comes from a happy home, but in reality, the animal may have been bred or kept in poor conditions.

To spot Petfishing, be vigilant for warning signs using the acronym

'SPOT':

- Seller – Put the seller's name and phone number into a search engine, avoid those with multiple adverts
- Parent – Make sure you see puppies and kittens in their home with their mother
- Old enough – Check puppies and kittens are at least eight weeks old before you take them home
- Treatment – Ask to see the animal's health records and avoid sellers who can't provide them

More information about the campaign is available at https://getyourpetsafely.campaign.gov.uk

Tools are available to help you identify a trusted breeder who prioritises the health and welfare of
the puppies they breed. The Puppy Contract is a free tool designed by the All Parliamentary Group for Animal Welfare, the Animal Welfare Foundation, the Blue Cross, the British Veterinary Association, DEFRA, the Dogs Trust, The Kennel Club, the PDSA and the RSPCA to help pet owners in their search for a healthy, happy puppy. For more information and a free downloadable version of the contract visit https://www.puppycontract.org.uk

There are also many puppies born into the puppy trade in the UK. If you think you may have come across a puppy farm, the best thing you can do is walk away and report it to:

- Trading Standards
- Police
- Local Authority of where the suspected puppy farm is
- RSPCA on 0300 1234 999 or online

Worried about your dog's health?
Please contact your dog's vet or register with a local vet if you have not registered your dog as yet. To find a local veterinary surgeon, please visit the following website: https://findavet.rcvs.org.uk/home/

Worried about your own mental health?
Puppy buying can be an emotional time and unfortunately things can go wrong causing owners heartbreak, guilt and worry. If you would like support or advice regarding your own mental

health, the following websites may be useful:
• https://www.nhs.uk/oneyou/every-mind-matters/
• https://www.samaritans.org

Worried about illegal importation?
If you are concerned animals you know of may have been illegally imported, the RSPCA advise you contact your local authority and/or call the RSPCA on 0300 1234 999.

Interesting in learning more about imported diseases?
This comprehensive document from the Animal Plant and Health Agency (2022) covers the major diseases which dogs imported from abroad, or travelling into the EU, risk bringing to the UK: https://tinyurl.com/4fhb8
